# Ergonomic Consideration of Sight Shifts between the Microscopic and Macroscopic Environments in Microscopic Dentistry for Inexperienced Operators

**DOI:** 10.3390/ijerph18157916

**Published:** 2021-07-27

**Authors:** Maciej Goczewski

**Affiliations:** Independent Researcher, ul. Okrzei 4, 83-000 Pruszcz, Poland; maciej@goczewski.pl

**Keywords:** dentistry, microscope, ergonomics, instrument transfer, eyes, view, sight

## Abstract

Due to the rising popularity of Dental Operating Microscopes, the issue of their ergonomic use has become an important topic. To verify its impact on dental teams’ ergonomics, the aspect of working in two distinct environments was examined. The research was performed on a group of 82 dentists regarding the number and character of shifts from the microscopic environment to the macroscopic one. The results revealed the shifts constitute a significant part of an inexperienced DOM operator’s work. Because such shifts, when performed repeatedly and in short succession, may influence the operator’s vision and comfort, they should be considered ergonomically detrimental. Having indicated the problem, the article puts forth a solution in the form of an instrument transfer methodology. The proposed adjustment has the potential of improving the quality of work for DOM operators quickly and efficiently by significantly reducing the number of shifts between the environments.

## 1. Introduction

The Dental Operating Microscope (DOM) has been a sign of technological advancement in dentistry for many years [1,2]. Yet the rise in its popularity has not been strongly reflected in the increase in research and systemic training material concerning ergonomics in microscopic dentistry. A review of the specialist literature reveals a lack of in-depth and well-founded practical solutions for dental teams and a scarcity of cohesive practical guidelines regarding the subject. Most information stems from non-peer-reviewed content prepared by individual practitioners and microscope manufacturers, such as the recent guide published by Zeiss [3]. Scientific literature, on the other hand, focuses on the aspect of posture and musculoskeletal issues, often presenting conclusions drawn from research in other areas characterised by a sitting work position [4,5].

Given the deficient guidance offered to DOM users, it is highly probable they perform microscopic dentistry based on their existing habits, instead of adjusting to the new conditions. The primary objective of this article is to verify this claim regarding the most prominent characteristic of utilizing the dental microscope—the necessity to work within two distinct environments, the macroscopic and the microscopic one.

Working without optical aids, that is in the macroscopic environment, feels natural. The operator can easily observe the whole area, utilizing a very wide view angle, and control all activities within but also around the operating area, if only by means of peripheral vision. Transitioning to the microscopic view poses a big challenge, as the view is extremely limited and separates the operator from any visual information from the surrounding area [5]. Many of the activities indirectly connected with the procedure, such as handling instruments and preparing materials, need to be relegated to the assisting personnel. Understandably, among the less experienced DOM users, there must be a strong incentive to switch from the microscopic environment to the macroscopic one during procedures, as this is their default mode of operation, their habit. If indeed such shifting takes place, its impact on the operator’s vision system should be considered. Firstly, it may lead to physical overload of the eye’s motor apparatus and secondly, induce sensory overload of the visual system.

Physical overload is typically induced by observing objects in a short distance, which is usually considered to be less than six meters, or 20 feet [6]. Under such conditions, maintaining proper vision and focus requires the eyes to pivot towards each other, and their lenses to contract. These two mechanisms, vergence and accommodation, rely on flexing and relaxing several groups of small muscles and, consequently, may lead to their overload [6,7]. It happens due to prolonged muscle contraction or repeated and excessive operation. Furthermore, the shorter the distance to the observed object, the more strain the tissue needs to withstand and the more work it performs to introduce the changes [5].

As the mechanisms adjust the visual system to the current conditions automatically and continuously, repetitive shifting from the microscopic environment, which simulates optimal viewing conditions and allows the muscles to relax, to the macroscopic one, where the observed area is usually within arm’s reach, forces the muscles to quickly change their state, exposing them to strain. This may lead to overloads and result in impaired vision, discomfort, and even pain [8,9].

Sensory overload, on the other hand, stems from quick changes in light intensity, when the eye is not given enough time to adapt to the new volume of photons reaching its surface [10]. The adaptation mechanism involves regulating the size of the iris and altering the chemical composition of photoreceptors to align the system’s sensitivity to the received light volume [6]. If the change occurs rapidly neither the iris nor the cells manage to prevent vision impairment. When moving from bright to dark conditions the system is desensitized and struggles to provide the brain with sufficient stimulus level, which means lowered colour perception, highly reduced visibility, or even total lack of vision. When moving in the opposite direction, the eye in its sensitive state becomes exposed to an overwhelming volume of light which causes a sensory overload resulting in temporary blinding and the appearance of visual artefacts.

The risk of the symptoms influencing the dental team stems from the work environment including two, three, or more distinctly lit environments [5]. As the microscope’s optical system absorbs a significant amount of light, the sources providing ambient lighting for the office are not sufficient for microscopic procedures. An additional lamp is required to compensate for the loss resulting in creating an intensely bright spot surrounding the operating area. This way two environments are created. Maladjustment of the lamp’s brightness results in an additional discrepancy between the micro and macroscopic environments, thus creating the third one.

In consideration of these mechanisms, it can be asserted that taking eyes off the microscope’s eyepiece repeatedly and in short succession may have an adverse influence over the visual system which is further implied by studies of other disciplines regarding human–machine interfaces [11]. Therefore, shifting between the micro and the macroscopic environments during a dental procedure should be considered ergonomically incorrect if repeated often, and in such a case, avoided.

Following this assumption, the research aimed at answering the question whether microscopic dentistry may subject its practitioners to frequent and rapid changes in visual environments, thus decreasing their work ergonomics and, if so, to propose a methodological solution for the problem. For this purpose a study of a group of students and young clinical practitioners was performed.

## 2. Materials and Methods

### 2.1. Subjects

The research was conducted between 2012 and 2019 on a total of 82 people aged 23 to 27. Ethical review and approval were waived for this study due to its observational character. Although both women and men were equally represented, the gender criterion was not considered within the study. What is more, no physical characteristics were considered, as the sole scope of the research was the operators’ behaviour with regard to environment shifts.

Based on preliminary surveys the subjects were divided into two groups based on their experience: Group A—students of the fourth and fifth year of dental studies (48 people) and Group B—graduates with no more than a year of dental practice (34 people). No subjects had received any substantial microscope training prior to the experiment. The aim of such division was to indicate if the initial stage of developing as a self-sufficient clinical practitioner, as opposed to being a student, is reflected in the frequency and type of environment shifts when utilizing a microscope.

### 2.2. Methodology

Each subject was presented with a randomly chosen assignment of filling a Class 1 lesion as classified by G. V. Black, or Si/Sta 1.3, according to classification by Mount and Hume [12], in modification by Lasfargues, Kaleka, and Louis [13]. Depending on the method chosen for the procedure, they were assigned to one of two groups: DF—direct field of view and IF—indirect field of view. The division was dictated by the increase in difficulty when working with a dental mirror (indirect field of view) due to lower accessibility of the operating field and the challenge of observing its reversed image. The more challenging work conditions might potentially influence the frequency and type of environment shifts when utilizing a microscope.

The participants were not informed of the aim of the research and were provided with the same dental office setup adjusted to the team of an operator and one assistant, with the microscope personalized to accommodate the operator, e.g., with regard to the interpupillary distance, diopter correction, or height. The microscope of the following specification and settings was used: Opmi Pico from Zeiss, focusing objective lens f = 250, 12.5×, widefield eyepieces, 180° tiltable tube, integrated video camera (full HD 1080 p) with three video displays for monitoring purposes, magnification changer factor = 0.4 (total magnification of 3.4× resulting in the field of view = 64 mm).

All procedures were recorded using two external cameras and the microscope-integrated video recorder simultaneously. A vocal cue was used for synchronization. The main camera utilized a wide angle to record the dental team, showing the operator, the assistant, the operating field, and the surroundings. The complementary camera, focused on the head of the operator, registered the more inconspicuous occurrences of shifting between the views. Both cameras were of the following setup: Sony NEX-VG20EH, resolution of 1920 × 1080, 50 frames per second.

The recordings were reviewed to count all occurrences of the operator looking away from the microscope. The occurrences were divided into several categories and classified based on specific cues, accompanying activities, and the typical order of particular steps within a dental procedure. The classification was done using the external recordings alongside the operating field view provided by the microscope camera. The following categories were distinguished:

C1—exiting the microscopic environment to control the tools within or directly next to the operating area (related to introducing the tool to the operating area, e.g., when the tool is beyond the view of the microscope and the operator needs to observe it directly to fix its position).

C2—exiting the microscopic environment to reach for instruments (e.g., when the operator exits the microscopic view when reaching for a tool to pick it up).

C3—exiting the microscopic environment to put away instruments (e.g., when the operator exits the microscopic view when putting a tool down to the instrument tray).

C4—exiting the microscopic environment to perform instrument transfer (e.g., when the operator exits the microscopic view when exchanging instruments with the assistant).

C5—exiting the microscopic environment to control the operating area and hand movement (e.g., when the shift occurs after the operator misplaces the instrument and corrects it while observing it directly, to revert to the microscope having done so).

C6—exiting the microscopic environment to make adjustments to the environment, such as correcting the position of the microscope or the footswitch (e.g., when the operator exits the microscopic view directly prior to repositioning the microscope head).

C7—exiting the microscopic environment to perform other activities indirectly connected to the procedure, such as applying and removing the composite or cleaning the mirror (e.g., when the operator exits the microscopic view directly prior to starting the activity, or a set of activities).

C8—exiting the microscopic environment for reasons irrelevant from the procedure’s standpoint, such as stretching, resting the eyes, or unspecified reasons (e.g., when the operator leaves the microscopic view and looks at the ceiling or the window).

C0—the categories C1 through C4 treated collectively as directly related to instrument transfer.

### 2.3. Statistical Evaluation

The results were collected and were subject to statistical analysis. For all tests, the chosen significance level of α = 0.05 was applied. The data were tested for the requirements of Student’s *t*-test. Due to the observations falling outside of the normal distribution, the Mann–Whitney *U* test was chosen to check for differences between the groups (A and B, DF and IF). The Bonferroni correction method was utilized in order to adjust the *p* values, controlling the family-wise error rate. Z-score was used to find the outliers distorting the results (Z-score > +2.5 SD or Z-score < −2.5 SD – SD: Standard Deviation). Spearman’s correlation coefficient was calculated to test the relationship between the number of instrument transfers and the number of shifts between the environments. No additional software was used to perform the calculations.

## 3. Results

After the Z-score analysis, nine subjects were removed from the dataset as outliers before the statistical tests were conducted.

Statistical analysis showed that shifting from the microscopic environment to the macroscopic one was a significant part of subjects’ work. The mean number of shifts per one procedure equalled 62.85 with a median of 60 (Table 1).

No statistically significant difference in the number of shifts between environments for students and graduates was found (Z = −0.43, *p* = 0.67, $p_{B}$ = 1.0, α = 0.05; $p_{B}$—Bonferroni-adjusted *p* value). Similarly, the groups working in the direct and indirect fields of view have been shown not to differ in this respect (Z = −0.51, *p* = 0.61, $p_{B}$ = 1.0, α = 0.05; $p_{B}$—Bonferroni-adjusted *p* value) (Table 2).

The most prevalent reason for taking the eyes off the microscope’s eyepiece was category C5, i.e., controlling the operating area and the hand movement. It represents 48.45% of all observed shifts, and on average it constituted 44.4% (SD = 0.16) of all occurrences per subject.

The second-highest number, and almost a third of all the shifts (29.8% of all shifts, with an average percentage of 31.3% per person, SD = 0.13), was related to instrument transfer, i.e., category C0, in which the activity of putting down the instruments was the most frequent (39.9% of the C0 category, and 39.2% on average).

No correlation was observed between the number of instrument transfers and the total number of shifts (Spearman’s correlation coefficient $r_{S}$ = −0.079, *p* = 0.51).

## 4. Discussion

### 4.1. Confirmation of the Environment Shifting Issue

**Figure A1:**
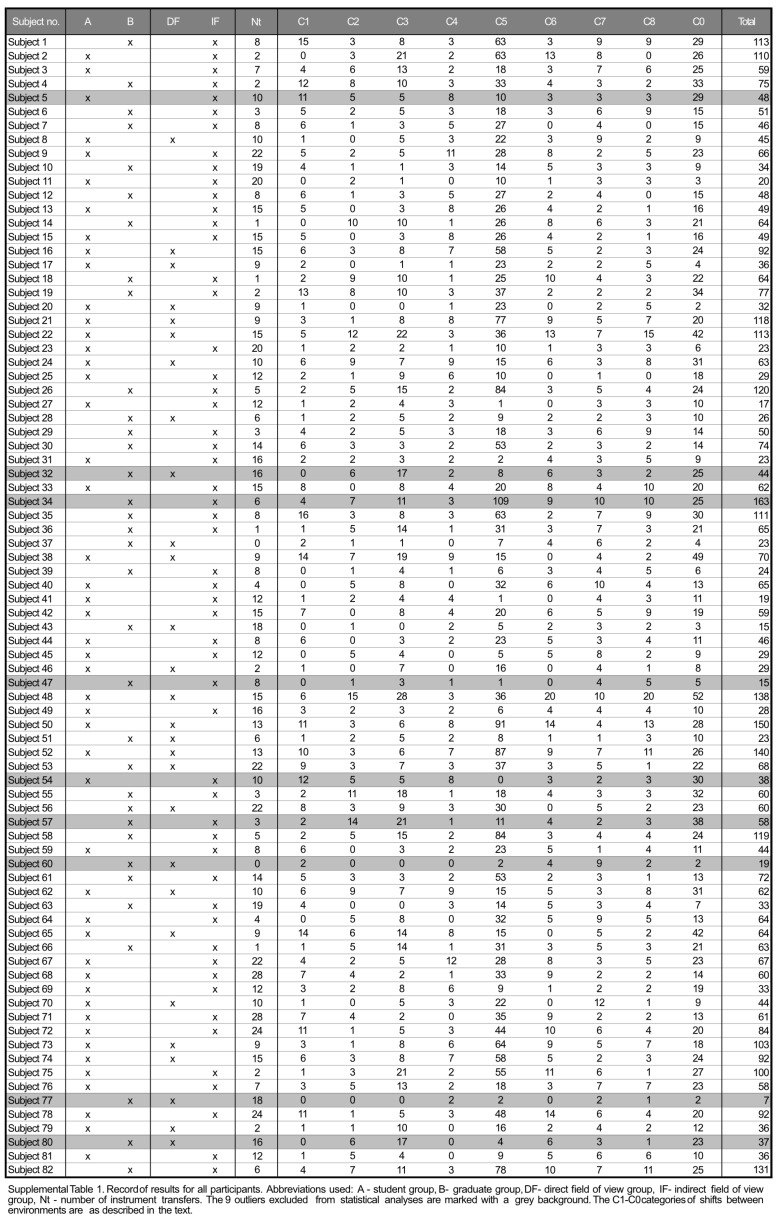
The results for individual subjects.

The results support the claim that the introduction of the Dental Operating Microscope to the practice does not force the dental team to adapt sufficiently to the new conditions. The dentists’ habit of observing the surroundings remained and they were leaving the microscopic view frequently and for short periods of controlling the operating area, glancing at tools, tracing hand movements, etc. The mean number of shifts of 62.85 (Table 1) and the extremity of 163 shifts (Figure A1) for a relatively simple procedure is arguably indicative of a problem, even if only regarding the efficiency of this procedure, which was purposefully disregarded in the research.

What is more, the lack of statistical differences between the two groups of different experience shows the problem is not countered by additional practice in the area of dentistry. The more experienced dentists have the same tendency to take their eyes off the eyepiece as students with very limited skill. Similarly, the lack of statistical differences between the two groups of different work methods shows that the problem does not lessen when easier procedures are performed, provided that the necessity of working in the indirect field of view increases the complexity of a procedure.

### 4.2. Countering the Problem of Shifting between the Environments

The indication of an extensive occurrence of environment shifts among novice DOM operators should be considered as a significant factor in dental team work ergonomics. As has already been explained, they are not only unnecessary but even potentially harmful if the recurrent shifts between the micro and macroscopic environments happen often and in short succession [11]. Therefore, the question arises of the frequency and period of such shifts which should be universally considered problematic—a question which in itself would constitute an interesting topic for further research. With the current state of knowledge in this respect, it is advisable to limit the shifts as a prevention measure decreasing the possibility of experiencing any detriment to the visual system.

Taking into account the prevalence of the C5 category within the study it would seem reasonable to target the tendency to observe the operating field and hand movement outside of the microscope’s field of view as the area with the biggest potential for reducing the number of shifts. Yet the C5 mistakes seem to find their roots in the lack of experience and not the lack of technical or methodological solutions. It may be claimed that with more work, as the dentist becomes more acquainted with microscopic dentistry and performs more challenging manual activities within the microscopic view, the problem should gradually subside. As reasonable as this is, it would require much time and training.

Yet, a third of the environment shift occurrences registered within the study happened for reasons directly related to work methodology—the instrument transfer. The only requirement for them to be reduced, or even eliminated, is to provide the operator with a convenient way of picking up tools, putting them away, introducing them to the operating area, and passing them to and from the dental assistants without leaving the microscopic view. This can be achieved by introducing a modified version of the transfer method of direct placement combined with parallel exchange suggested by Valachi for over-the-patient and transthoracic delivery systems [14].

### 4.3. A Proposition of Work Methodology Addressing the Problem of Environment Shifts

The main assumption of this work methodology is that the operator focuses entirely on the operating area while the assisting personnel handles all the tools outside of the immediate scope of the procedure. This means the instruments are handed to the operator and returned to their place by assistants, who additionally control the transfer and introduce instruments to the operating field by guiding the operator’s hand. This way the main reasons for leaving the microscopic environment are removed and the procedure can be performed without unnecessary strain to the visual system. The transfer method can be performed with the same effectiveness for all kinds of instruments, static and dynamic alike, for both left and right hands, and in either four-handed or six-handed dentistry, provided a proper preparation of the dental team occurs. It includes the following stages: 

PREDELIVERY

Predelivery is the moment of anticipating the transfer and it involves the assisting personnel only. As the operator focuses on the procedure itself, the assistant is required to track the progress of the operation and predict when a change in tools is going to happen. To prepare for the transfer the following steps are taken:

1. The assistant holds the instrument by its non-working end, positioning it parallel to the instrument held by the operator.
2. When the transfer approaches, the assistant moves towards the instrument that needs to be removed from the operator’s hand.

EXCHANGE

Exchange is the activity of switching tools during the procedure. It means that the assistant takes the unnecessary tool away from the operator’s hand and replaces it with a different one. The steps are:

1. The operator retracts the currently used instrument from the operating area and moves it slightly away, towards the assistant.
2. Carrying the new tool in the main fingers, following the predelivery setup, the assistant uses the remaining, unoccupied fingers of the same hand to remove the exposed instrument from the operator’s grasp.
3. With two instruments held in one hand, the assistant rotates the wrist to place the new one in the operator’s grasp.

PLACEMENT

Placement is the most significant of the stages if unobstructed microscopic observation of the operating field is considered. It is the action of introducing the new tool to the operator’s hand, but also allowing the operator to move it inside the microscope’s field of view. It is achieved through the “mutual grasp” of the tool. The stage is performed mostly as a continuation of the exchange stage but may also occur in the so-called direct placement, which refers to the situation when the operator is not holding any instruments when the transfer begins. Its steps are:

1. The assistant transfers the instrument, putting it into the operator’s hand, positioning it in such a way that it can be taken with the proper grasp, such as the two-finger, the palm, or the pen grasp while requiring as little adjustment on the operator’s part as possible.
2. Having felt the instrument’s touch the operator takes it with the grasp appropriate to the tool’s characteristic and own preference.
3. At this point both the operator and the assistant keep hold of the same tool which allows the assistant to introduce the instrument to the microscope’s field of view, at the same time guiding the operator’s hand to the proper position at the patient’s oral cavity.
4. The operator obtains the view of the tool and with it the ability to continue the procedure. At this point, the assistant can safely let go of the instrument and retract the hand, while the operator introduces the tool to the operating area.
5. The transfer is finished and the procedure continues.

In the case of two instruments being transferred at the same time, the assistant may proceed to hand over the second tool after the first tool has been taken by the operator. The same steps should be followed for both hands.

It might also be assumed that returning the unused tool to its original location, e.g., the tool tray or the dental unit console is also an indispensable part of this process and should be considered the last step of the transfer procedure, as held by Bladowski [15].

## 5. Conclusions

The research has shown that inexperienced microscope users exhibited a significant level of shifts between the two environments. Choosing unaided observation to such an extent even during a relatively simple procedure is concerning and poses a clear indication of ergonomic flaws in the assumed methodology. Further research would be advisable to compare the results with those of a group of experienced dentists with little microscope experience and experienced microscope operators.

Although the topic of frequent changes in observed environments has not been covered in the specialist literature well enough for a consensus to arise in the matter of its detriment to the visual system, the body of literature on other occupations, their ergonomics, and work conditions, together with an in-depth analysis of the human eye structure and operation does support forwarding a reduction in the number and frequency of such shifts.

A solution addressing the issue and eliminating a large portion of shifts systemically is the implementation of a particular methodology of instrument transfer, facilitating continuous observation of the operating field and entrusting the dental assisting personnel with controlling the macroscopic environment of the dental procedure.

## Figures and Tables

**Table 1:** The total number of shifts from the microscopic environment to the macroscopic one for all categories, 9 outliers not included.

Category	Total	Mean	Median	Total%	Average%
C1	332	4.55	4	7.2%	7.2%
C2	244	3.34	2	5.3%	5.6%
C3	544	7.45	6	11.9%	12.3%
C4	245	3.36	3	5.3%	6.2%
C5	2223	30.45	25	48.5%	44.4%
C6	346	4.74	4	7.5%	7.6%
C7	332	4.55	4	7.2%	8.9%
C8	322	4.41	3	7.0%	8.0%
C0	1365	18.70	18	29.8%	31.3%
SUM	4588	62.85	60	n.a.	n.a.

*n* = 73.

**Table 2:** The combined number of shifts per studied group for all categories, 9 outliers not included.

Category	A *n* = 46	B *n* = 27	DF *n* = 24	IF *n* = 49	SUM
C1	201	131	118	214	332
C2	139	105	85	159	244
C3	347	197	196	348	544
C4	182	63	104	141	245
C5	1324	899	785	1438	2223
C6	254	92	114	232	346
C7	212	120	112	220	332
C8	220	102	128	194	322
C0	869	496	503	862	1365
SUM	2879	1709	1642	2946	4588

A—student group, B—graduate group, DF—direct field of view group, IF—indirect field of view group, *n*—number of subjects.

## Data Availability

All the data used for analysis has been provided in Appendix A.
